# Long Non-Coding RNAs: The Key Players in Glioma Pathogenesis

**DOI:** 10.3390/cancers7030843

**Published:** 2015-07-29

**Authors:** Karrie Mei-Yee Kiang, Xiao-Qin Zhang, Gilberto Ka-Kit Leung

**Affiliations:** Department of Surgery, Li Ka Shing Faculty of Medicine, The University of Hong Kong, Queen Mary Hospital, Hong Kong, China; E-Mails: kkarrie@hku.hk (K.M.-Y.K.); zhxq@hku.hk (X.-Q.Z.)

**Keywords:** lncRNA, glioma, CRNDE, HOTAIR, H19, MEG3, glioblastoma stem cell, biomarker, gliomagenesis

## Abstract

Long non-coding RNAs (LncRNAs) represent a novel class of RNAs with no functional protein-coding ability, yet it has become increasingly clear that interactions between lncRNAs with other molecules are responsible for important gene regulatory functions in various contexts. Given their relatively high expressions in the brain, lncRNAs are now thought to play important roles in normal brain development as well as diverse disease processes including gliomagenesis. Intriguingly, certain lncRNAs are closely associated with the initiation, differentiation, progression, recurrence and stem-like characteristics in glioma, and may therefore be exploited for the purposes of sub-classification, diagnosis and prognosis. LncRNAs may also serve as potential therapeutic targets as well as a novel biomarkers in the treatment of glioma. In this article, the functional aspects of lncRNAs, particularly within the central nervous system (CNS), will be briefly discussed, followed by highlights of the important roles of lncRNAs in mediating critical steps during glioma development. In addition, the key lncRNA players and their possible mechanistic pathways associated with gliomagenesis will be addressed.

## 1. Introduction

The large repertoire of non-protein-coding RNAs found in transcriptional output has intrigued and inspired scientists about the fundamental functions of these genomic sequences, and has become a significant milestone in non-coding RNAs (ncRNAs) research [1,2]. As early as 1980s, RNA transcripts without significant protein-coding potentials were examined in *Drosophila* [3]. It was shown that these transcripts had genetic effects and were all spatial-temporally regulated. Moreover, interruptions of the encoding DNAs were found to cause phenotypic changes in *Drosophila*. This diverse subgroup of RNA species were later identified also in human as ncRNAs that have no protein-coding ability, and were shown to function intrinsically at the RNA level [4,5]. Over the evolutionary course of time, it was found that some ultraconserved elements in *Drosophila* were located within the non-coding regions of its genome [6]. Alterations of these ncRNA-associated ultraconserved sequences could lead to fatal diseases including cancers [7]. Although these ncRNAs do not code for proteins [8,9], they appear to have structural and regulatory roles that are important for normal cellular function as well as disease pathogenesis [10]. NcRNAs generally lack open reading frames. In terms of structure, long non-coding RNAs (lncRNAs) differ from other RNA species such as miRNAs, snoRNAs, siRNAs, piRNAs, snRNAs and tRNAs, in that the former are longer than 200nt while the latter group encompasses transcripts shorter than 200nt [2,11,12,13,14,15].

Gliomas are the most common form of primary malignant brain tumor, and glioblastoma multiforme (GBM) is the most aggressive form of glioma with a median survival of 15 months following standard treatment [16,17]. GBM cells are known to carry multiple molecular and genetic aberrations [18]. For example, methylation of the promoter of the DNA repair enzyme, *O*-6-methylguanine-DNA methyltransferase (MGMT), is found in about 50% of all GBM cases [19]. MGMT promoter methylation silences its expression, and eventually limits the tumor’s ability to repair DNA breakages following temozolomide. This enhances the cytotoxic effects of temozolomide and thus treatment efficacy [20,21]. Although the current multimodal treatment regime with surgical resection, concurrent chemoirradiation and adjuvant temozolomide has provided significant improvement in patient survival, tumor recurrence occurs in most if not all cases [22,23,24]. As a rescue therapy, bevacizumab, an antiangiogenic drug, is often used in clinical practice upon tumor recurrence [25]. However, bevacizumab can only slow down recurrent tumor growth without exerting any beneficial effects on overall survival [26].

NcRNAs have recently emerged as potentially promising therapeutic targets in cancer therapy. Various experimental approaches, including direct RNA sequencing, cloning, microarray and genomic SELEX (genomic systemic evolution of ligands by exponential enrichment), have been developed for the studying of RNomics [27]. Based on microarray-based data, we have previously shown that specific lncRNA expression patterns were associated with different histological subtypes and malignant behaviors in glioma [28]. Furthermore, certain lncRNAs were found to be of prognostic significance, suggesting that lncRNAs may have important roles in gliomagenesis and may serve as novel therapeutic targets and biomarkers [29]. In this review, we discuss the functional aspects of lncRNAs within the central nervous system (CNS) and their roles in glioma pathogenesis. In addition, the key lncRNA players and their possible mechanistic pathways associated with gliomagenesis will be addressed.

## 2. Overview on lncRNAs

It has now become clear that lncRNAs are involved in various genetic phenomena, including imprinting, DNA methylation, X-chromosome dosage compensation as well as transcriptional, post-transcriptional and epi-genetic regulations [9,30,31,32,33,34]. Mounting evidence has also demonstrated that lncRNAs may regulate gene expressions through interactions with DNAs, RNAs, proteins or chromatin remodeling [35,36]. In recent years, new bioinformatical and experimental strategies have been established which allow the identification of a large number of novel lncRNA transcripts. With the aids of current biocomputational research tools such as lncRNAdb, ChIPBase, LNCipeida and lncRNAtor, the number of lncRNAs being identified is rapidly increasing [37,38,39,40]. These tools have also provided very useful platforms for biophysical analyses that can predict lncRNA interactions with other genomic elements.

The diverse transcription patterns of lncRNAs have significant implications for their gene regulatory functions. LncRNAs can be expressed in intergenic or intronic regions, or in overlapping or antisense loci adjacent to protein-coding genes, on which lncRNAs may exert regulatory functions [41,42,43]. Gene expression may also be regulated through lncRNAs’ interactions with chromatin modifying complexes. Many studies have demonstrated lncRNA-EZH2 interaction. EHZ2 is an enhancer of zeste homolog 2 (a predominant component of chromatin modifying protein PRC2, polycomb repressive complex 2)-dependent tumor suppressive/oncogenic activities. This association may also serve to guide these chromatin modifying complexes to the target loci [44,45,46]. There is accumulating evidence suggesting that the association of lncRNAs with EZH2 is implicated in cancer biology through up/downregulation of gene expressions [47,48]. Compared to protein-coding genes, lncRNAs are highly tissue-specific, and are often co-expressed with neighboring coding genes [49]. Novel functions of lncRNAs are steadily emerging. More recent findings suggested that lncRNAs may in fact affect protein-coding directly. It was shown that, instead of being localized within the nucleus, the majority of lncRNAs were found within the cytoplasm in association with ribosomes where they may serve as repositories for the evolution of new protein species [50,51].

As suggested by the hypothesis of competitive endogenous RNA (ceRNA), it attributes novel function of lncRNAs in the “communication” network across the RNA transcriptome, through microRNA binding sites (or microRNA response elements—MREs) covered within RNAs [52]. Current experimental evidences are in support with this hypothesis [53,54,55,56], whereas pseudogenes and/or lncRNAs forms a ceRNA network of RNA crosstalk by acting as molecular sponges for microRNAs thereby modulates its gene repressive activity. This ceRNA activity implicates a novel ceRNA function of lncRNAs, and perturbation of ceRNA might have consequences in pathological conditions including cancer [53,57].

## 3. Functional Roles of lncRNAs within the CNS

Of the tens of thousands of lncRNAs so far identified, relatively few have been functionally tested and their precise roles in the diversity of cellular processes remain unknown [14,58]. Since lncRNAs are predominantly expressed within the CNS [14,58,59] and are spatial-temporally regulated during development [34,60], they are now thought to serve important functions in CNS development while perturbations of their expressions may lead to various CNS pathologies [60,61,62]. Many lncRNAs exhibit specific expression profiles in distinct neuroanatomical regions, and are associated with specific cell types and subcellular compartments [61]. This presence of a huge proportion of lncRNAs within the brain may underlie their complexity and sophistication as compared to the case in other tissue types [14,63].

In a functional analysis of evolutionarily conserved intergenic lncRNAs in mouse, “brain clusters” of lncRNAs were identified [64]. These were found to be differentially expressed during developmental transitions, indicating that lncRNAs may mediate crucial functions in neural differentiation [65]. One functional study performed by Sauvageau *et al*. has provided strong evidence that lncRNAs, in particular BRN1B, are critical for life, organ and brain development *in vivo* using several lncRNA knockout models [66]. Many other studies on lncRNA ablation also suggested that certain lncRNAs are required for normal brain development although loss of function would result in only subtle phenotypic abnormalities, if any [67,68,69,70]. LncRNAs also play roles in determining neural cell fate. This is partly mediated through the lncRNA Sox2OT, a counterpart of the important stem cell regulator gene Sox2 [71]. Another lncRNA, Nkx2.2AS, was shown to be critical for the lineage differentiation of oligodendrocytes during neural stem cell (NSC) differentiation [72]. As mentioned, dysregulation of lncRNAs may cause brain malformations, and are closely linked to the pathophysiology of various CNS diseases such as Down’s syndrome, Alzheimer’s disease, multiple sclerosis, brain tumor and schizophrenia [60]. Some of the known lncRNAs associated CNS diseases are summarized in Table 1.

**Table 1:** LncRNA-associated diseases in the CNS.

LncRNA	LncRNA-Associated CNS Diseases	References
Ube3a-as	Neurodevelopmental disorder—Angelman syndrome	[73]
DGCR5	Neurodevelopmental disorder—Velocardiofacial syndrome	[74]
NRON	Neurodevelopmental disorder—Down’s syndrome	[75]
BACE1-AS	Neurodegenerative disorder—Alzheimer’s disease	[76]
BC200	Neurodegenerative disorder—Alzheimer’s disease	[77]
Tmevpg1	Neuroimmunological disorder—Multiple sclerosis	[78]
H19	Neurooncological disorder—CNS tumors	[79]
DISC2	Psychiatric disorders	
	Schizophrenia, bipolar disorder, depression, autistic spectrum disorder	[80,81,82]

## 4. LncRNAs in Glioma

Given that numerous lncRNAs are involved in a wide range of CNS pathophysiology, it has generally been accepted that they may also be key regulators in brain cancers. Genome-wide profiling studies have revealed differential expression patterns of lncRNAs in normal and cancerous tissues as well as across different cancer types [83,84,85,86]. Information on the central role of lncRNAs in gliomagenesis has only become clearer during the past few years. LncRNAs appear to be exceptionally important in all different aspects of glioma pathophysiology, from malignant transformation to tumor recurrence, and also in disease prognosis.

### 4.1. Glioma Initiation, Progression and Recurrence

In the context of cancer initiation and transformation, lncRNA expression profiles between normal brain tissue and gliomas are significantly different. Certain lncRNAs are involved in cancer progression, and gliomas of different malignancy grades have also been shown to have differential lncRNA expressions [28,87,88]. One example is H19. Its expression is highly upregulated in gliomas. It can bind with transcription factor c-Myc to drive tumor transformation and contribute to tumorigenic phenotypes [89]. The expressions of H19, MALAT1 and POU3F3, for instance, were positively correlated with more malignant glioma phenotypes, and H19 also modulates glioma cell invasion [88,90,91]. Han *et al*. has also described the role of lncRNAs in glioma recurrence. Through comprehensive pathway analysis, the PPAR signaling pathway was found to be the most significant pathway through which glioma-associated lncRNAs may act [85]. Analyses on lncRNA-gene network in this pathway indicated that both ASLNC22381 and ASLNC20819 would target IGF-1, which is strongly implicated in glioma recurrence [85,92]. Currently, there is no evidence showing any correlation of lncRNAs in brain tumor metastasis, which is, afterall, relatively uncommon due to the impermeable nature of the blood brain barrier [93]. In spite of this, HOTAIR has been demonstrated in promoting metastasis in other cancer types via the modulation of epigenome [94].

### 4.2. Glioma Classification and Prognostication

Profiling studies on lncRNAs have important clinical implications for glioma subclassification as well as disease prognostication. LncRNA-based molecular subclassification by Li *et al*., has revealed three distinct subtypes of glioma. More specifically, they can be classified into lncRNA signature subgroups: (i) astrocytic tumor with high EGFR amplification; (ii) neuronal-type tumor; and (iii) oligodendrocytic tumor enriched with IDH1 mutation and 1p19q co-deletion. This lncRNA-based classification was found to be strongly correlated with patient survival [95]. Furthermore, an analysis on previously published microarray data has explored a six-lncRNA signature as a set of prognostic genes in glioma. PART1, MGC21881, MIAT, GAS5 and PAR5 were correlated with prolonged survival, while KIAA0495 was associated with poorer survival [29]. In another study, MALAT1 expression was shown to be elevated in glioma tissues when compared with adjacent normal brain tissue; increased expression was correlated with poorer overall patient survival [91]. HOTAIR expression level was also identified as another strong prognostic factor [96].

### 4.3. LncRNAs in Glioma Stem Cells (GSCs)

It has been proposed that GSCs possess much greater tumorigenic potential than their “non-stem” counterparts [97,98], and that GSCs are relatively resistant to radiation as well as chemotherapies [99]. The functional role of lncRNAs in GSCs has been demonstrated in a recent comparative analysis of microarray data. In this, glioma lncRNA expressions from several different stemness-related datasets were examined. Within the same tumor bulk, subpopulations of tumor cells were derived from their parental GBM cells based on differentiation status and surface marker CD133+ expression. These subpopulations were found to possess different patterns of lncRNA expression, such as the upregulations of H19, XIST and MIAT in undifferentiated tumor cells. In another dataset, lncRNAs H19 and HOTAIR expressions were also dysregulated in CD133+ subtype as compared to CD133- cells. The author also compared between GSCs and NSCs, and found relatively upregulated HOTAIRM1 and H19 expressions in the former. These results strongly implicate the role of lncRNAs in the maintenance of stemness and tumor propagation. A more detailed review on lncRNA in GCSs has been described by Zhang *et al*. [100].

## 5. LncRNA Dysregulation in Glioma

LncRNAs are involved in many biological processes in glioma cells, including cell proliferation, apoptosis and invasion [68,90,101]. Aberrant expressions of lncRNAs in gliomas have been reported extensively in genome-wide studies [36,85], and are potentially implicated in determining glioma development through interaction with different molecules and through diverse signaling pathways. For example, MEG3 controls proliferation via interacting with p53 and MDM2 protein [102]; CRNDE regulates glioma cell growth via mTOR signaling [103]; and ASLNC22381 and ASLNC20819 promote proliferation through the IGF-1R signaling pathway [104].

**Figure 1:**
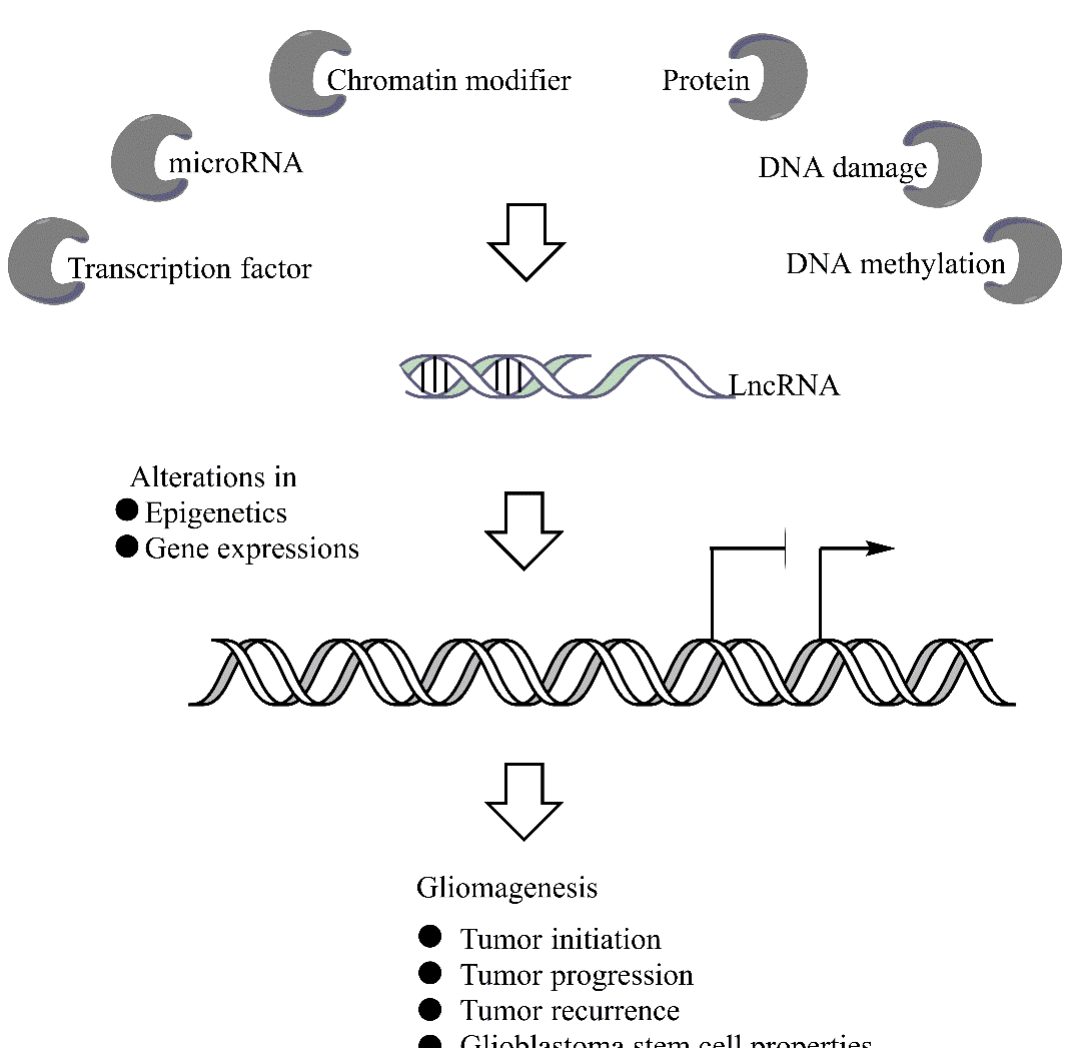
Gene regulatory network of lncRNAs in glioma oncogenesis. Different molecules and various cellular conditions are able to regulate lncRNAs expressions. From which dysregulations of lncRNA would cause pro-tumorigenic alterations in epigenetics and/or global gene expressions that promotes glioma development and the associated malignant phenotypes.

However, the mechanisms through which lncRNAs regulate signaling pathways remain largely unknown. Figure 1 illustrates the regulatory networks of lncRNAs that have been reported so far. It has been proposed that dysregulation of lncRNAs are particularly associated in glioma pathogenesis [105]. One mechanism is, for instance, the transcriptional regulation by transcription factors (TF). Biocomputational analyses have demonstrated abundant TF binding sites in lncRNA promoter regions [38,100,106]. Moreover, TF could bind directly to lncRNAs and regulate their expressions. Ma *et al.* showed that HOTAIR is a direct target of TF c-Myc, by which HOTAIR is activated and can drive tumor progression [106]. c-Myc can induce H19 expression and may play an important role in tumor transformation [89]. LncRNA expressions are also regulated by epigenetic changes through DNA hypo/hypermethylation [107].

Under stress conditions induced by genotoxic agents, changes in lncRNA expression may occur in response to DNA damage in glioma cells. In particular, MEG3 and ST7OT1 were upregulated during genotoxic stress-induced cell death; TUG1, BC200 and MIR155HG were downregulated [108]. These suggest that distinct pathways of lncRNAs are regulated in response to different conditions. LncRNA gain- and loss-of-function studies showed that these responses may have specific oncogenic or tumor suppressive functions [88,105,106,109,110]. A numbers of lncRNAs have been consistently found to be dysregulated in glioma, and which are extraordinarily associated with malignant transformation. Here, we will discuss the key lncRNAs as mediators in glioma pathogenesis.

## 6. Examples of Well-Characterized lncRNAs in Glioma

### 6.1. H19

H19 was the first lncRNA reported as a tumor suppressor in mammalian cells in 1993 [111,112]. Hao *et al.* suggested an anti-tumorigenic effect of H19 following the observation that ectopic expression of H19 would retard embryonic tumor growth. In addition, both clonogenicity and tumorigenicity were compromised *in vivo* with the addition of H19 constructs [111]. Interestingly, recent analyses showed inconsistent patterns of expressions in several human cancers [113,114]. Abundant binding sites for TF c-Myc has been revealed in H19 promoter region [100]. Upon direct binding of c-Myc to H19, H19 gene transcription was significantly induced through histone acetylation in tumor cells [89]. On the other hand, several studies reported that H19 expression was positively correlated with glioma grading and that its expression is critical in tumor progression as well as invasion [90]. H19 is one of the most highly expressed lncRNAs in the placenta and was found at high levels particularly during embryonic development within endodermal and mesodermal embryonic tissues. Its expression level would become relatively downregulated after birth [115,116]. As such, H19 expression has been functionally implicated in the maintenance of stemness in hematopoietic/embryonic stem cells [117,118]. Consistent findings are also seen in the context of GSCs, as H19 is one of the most highly upregulated lncRNAs in GSCs as compared to its differentiated counterparts of glioma cells [100]. To date, the underlying role and mechanisms by which H19 may affect glioma development and in GSCs remain unclear.

### 6.2. HOTAIR (HOX Transcript Antisense Intergenic RNA)

As a well-recognized lncRNA, HOTAIR primarily serves as a negative prognostic gene in different cancers including GBM [119]. The expression patterns of HOTAIR are closely associated with glioma staging, and its increased expression with tumor progression [96]. In addition to lncRNA profiling, Pastori *et al*. performed a single molecule sequencing (SMS) expression analysis that robustly identified differential patterns of lncRNA alterations in GBMs. HOTAIR was found to be highly upregulated in GBM cells compared with control, and glioma cell growth was significantly reduced following depletion of HOTAIR transcript [86].

Functional studies have demonstrated that loss of HOTAIR would render glioma cells more susceptible to cell-cycle arrest, with retarded tumor growth and reduced tumor cell invasiveness [96,119]. In glioma, HOTAIR expression can be activated in c-Myc targeted transcription, which has been shown to drive tumor progression while suppressing miRNA-130a expression [106]. The pro-oncogenic activity of HOTAIR may also be mediated through direct binding to its target chromatin modifying complexes PRC2. As a result of this interaction, histone H3K27 is trimethylated, leading to epigenetic silencing of gene expression [120].

Given the role of HOTAIR in epigenetic regulation through PRC2, inhibition of the bromodomain and extraterminal (BET) proteins may exert antiproliferative effect on GBM cells while reducing HOTAIR expression. Together with the observation that BET proteins could bind directly to HOTAIR promoter, these findings strongly suggest that BET protein may regulate cell proliferation at least partly through HOTAIR [86]. Despite mounting evidence suggesting the oncogenic role of HOTAIR in glioma, the mechanisms by which it regulates gene expression is incompletely understood.

### 6.3. CRNDE (Colorectal Neoplasia Differentially Expressed)

CRNDE was firstly identified as a novel lncRNA biomarker for colorectal cancer, in which its expression is highly upregulated [121,122,123]. Over 90% of colorectal adenoma and adenocarcinoma displayed elevated expressions of CRNDE compared to normal colorectal tissue in a microarray study. Strikingly, it was found that individual CRNDE transcript isoforms, CRNDE-h, could be detected in patient plasma with promising value as a biomarker [123]. Indeed, CRNDE expression is also overexpressed in many other cancers including glioma [28,123,124,125]. It is found to be the most upregulated lncRNAs in GBM, with a 32-fold increase over that in normal brain tissues. Results from the same study also indicated that CRNDE expression level was closely associated with glioma grading [28]. Forced overexpression of CRNDE have resulted in increased glioma cell growth and migration, while knockdown of CRNDE would suppress oncogenic activities [103].

Similar to the case of HOTAIR, binding of chromatin modifying complexes CoREST and PRC2 to CRNDE suggested that CRNDE may regulate gene expression via epigenetic changes of histone methylation/demethylation [126]. The progressive loss of CRNDE expression from birth is suggestive of a tentative link between CRNDE expression and cell differentiation. This notion is supported by the observation that CRNDE is required for maintaining pluripotency of mouse embryonic stem cell (moESC). The binding of pluripotency-related transcription factor to CRNDE transcript has provided evidence of CRNDE as a target of the stemness pathway [127]. On the other hand, EGFR expression has been linked to GSC phenotype, and may contribute to the aggressive behavior of tumor-initiating cells [128,129]. In consistent with these findings, CRNDE-expressing gliomas were found to have EGFR over-amplification, suggesting that, to a certain extent, CRNDE may be involved in the regulation of GSCs through the EGFR signaling pathway [130].

### 6.4. MEG3 (Maternally Expressed Gene 3)

Unlike most of the lncRNAs mentioned earlier in this article that possess pro-oncogenic properties, MEG3 represents a tumor suppressor lncRNA that is associated with prolonged survival in GBM patients [28,101,131,132]. It has been found to be highly expressed in normal brain tissue and downregulated in gliomas [28,101]. Glioma cell proliferation was inhibited with increased apoptosis when MEG3 was overexpressed [101]. This anti-proliferative function is exerted, in part, through suppressing MDM2 and the subsequent activation of p53 signaling pathway [102,132]. The MEG3 knockout mice model generated by Gordon *et al*. has revealed the functional role of MEG3 in regulating vascularization in the brain. An increase in microvessel formation was seen in the brains of MEG3-null embryos, together with elevated expression of genes involved in VEGF angiogenic pathway. Besides, lost expression of MEG3 was observed in the majority of clinically non-functioning pituitary adenomas [68,131]. Taken together, the potential implication of MEG3 as a therapeutic target in the treatment of glioma is considerable.

## 7. Clinical Implications of lncRNAs in Glioma

The discovery of this novel class of RNA transcripts has provided valuable insights into their exploitations as therapeutic targets. Differential expressions of lncRNAs between normal and different grades of gliomas offer significant promises of using lncRNA signatures in glioma diagnosis and prognostication. Some lncRNAs are detectable in body fluids of cancer patients, for example, DDA in urine samples of prostate cancer patients, and CRNDE in the plasma of colorectal cancer patients [123,133,134]. This provides a non-invasive method for the assessment of disease progression. Given that cancer stem cells contribute significantly to treatment resistance, it can be postulated that GSCs-associated lncRNAs would appeal as attractive targets for more effective cancer medicine in combating recurrent diseases [99]. For instance, epigenetic modulator proteins targeting GBM-specific lncRNAs may potentially restore the normal epigenetic landscape and provide clinical benefit [86].

## 8. Conclusions

LncRNAs are abundantly expressed in the brain compared to other regions. However, the vast majority of lncRNAs in the brain have not yet been functionally characterized. With extensive efforts, some of them are now emerging as important players in glioma and GSCs. It has been a rapid development in uncovering the functional roles of lncRNAs over the past few years although many of the studies are based on bioinformatics analyses without *in vivo* evidence. Several regulatory mechanisms have been proposed that might contribute to the dysregulation of lncRNAs, while its aberrant expressions are thought to increase the propensities of tumor development. Besides, the interactions of lncRNAs with different molecules have posited the formers’ roles as mediators in key signaling pathways, thus regulating global gene expression and affecting a wide range of cellular processes. In spite of all these progresses, the regulatory network of lncRNA in glioma remains largely elusive. We believe that future investigations will eventually give rise to fruitful clinical translations of glioma-associated lncRNAs profiling into novel therapeutic paradigms.
